# High Seroprevalence for *Rickettsia rickettsii* in Equines Suggests Risk of Human Infection in Silent Areas for the Brazilian Spotted Fever

**DOI:** 10.1371/journal.pone.0153303

**Published:** 2016-04-11

**Authors:** Celso Eduardo Souza, Luciana Bonato Camargo, Adriano Pinter, Maria Rita Donalisio

**Affiliations:** 1 Reference Rickettsial Diseases Laboratory, Superintendence for Control of Endemic Diseases, São Paulo, Brazil; 2 Department of Public Health, State University of Campinas, São Paulo, Brazil; University of Texas Medical Branch, UNITED STATES

## Abstract

Equines play a role in the epidemiology of Brazilian spotted fever (BSF) since they are a primary host for the tick *Amblyomma sculptum*. We studied the seroprevalence for three species of Rickettsia in equines in four endemic (with human cases) and in four non-endemic areas (no human cases) in the Piracicaba River Basin, São Paulo, Brazil. A serological survey of 504 equines was performed: around 63 animals were sampled in each area and tested through indirect immunofluorescence assay for *R*. *rickettsii*, *R*. *parkeri*, and *R*. *bellii* in 2012–2013. Blood samples were seropositive for 183 equines (36.3%) in which 73 (39.9%) were from non-endemic areas. In the studied sites equines were highly exposed to Rickettsia infection ranging from 6.1% to 54.7%, with Geometric Mean Titers greater in endemic area (p = 0.012). Results suggest that Rickettsia may be more widespread than the surveillance of BSF has detected. These results highlight the need to include data on the seroprevalence of sentinel animals to improve human diagnoses and surveillance in areas with no reported human cases.

## Introduction

Brazilian spotted fever (BSF) is the most important rickettsial disease in Brazil [1]. BSF is an acute infectious disease whose etiological agent *Rickettsia rickettsii* is transmitted to humans and to other animals by several different tick’ species, including *Amblyomma sculptum*, the main vectors of BSF in the country. Capybara (*Hidrochoerus hidrochaeris*) is one of the primary hosts of *A*. *sculptum* and is particularly abundant in endemic areas for BSF [2]. Capybaras were reported as a competent amplifier host for *R*. *rickettsii* and the most important tick host in Spotted Fever transmission areas where *A*. *sculptum* is the vector to humans [3]. Other wild animal tested as an amplifier hosts was the opossum (*Didelphis* sp.) which showed a minor competence [4] [5].

Horses are one of the main element of connection in the epidemiology of Rickettsia in Brazil because they are primary hosts of all stages of *A*. *sculptum* tick. In addition, since horses can travel across large distances and are frequently used for transport in rural settings, they can also act as an agent to disperse infected ticks to other areas causing the emergence of a new focus of infection [6] [7].

**Fig 1:**
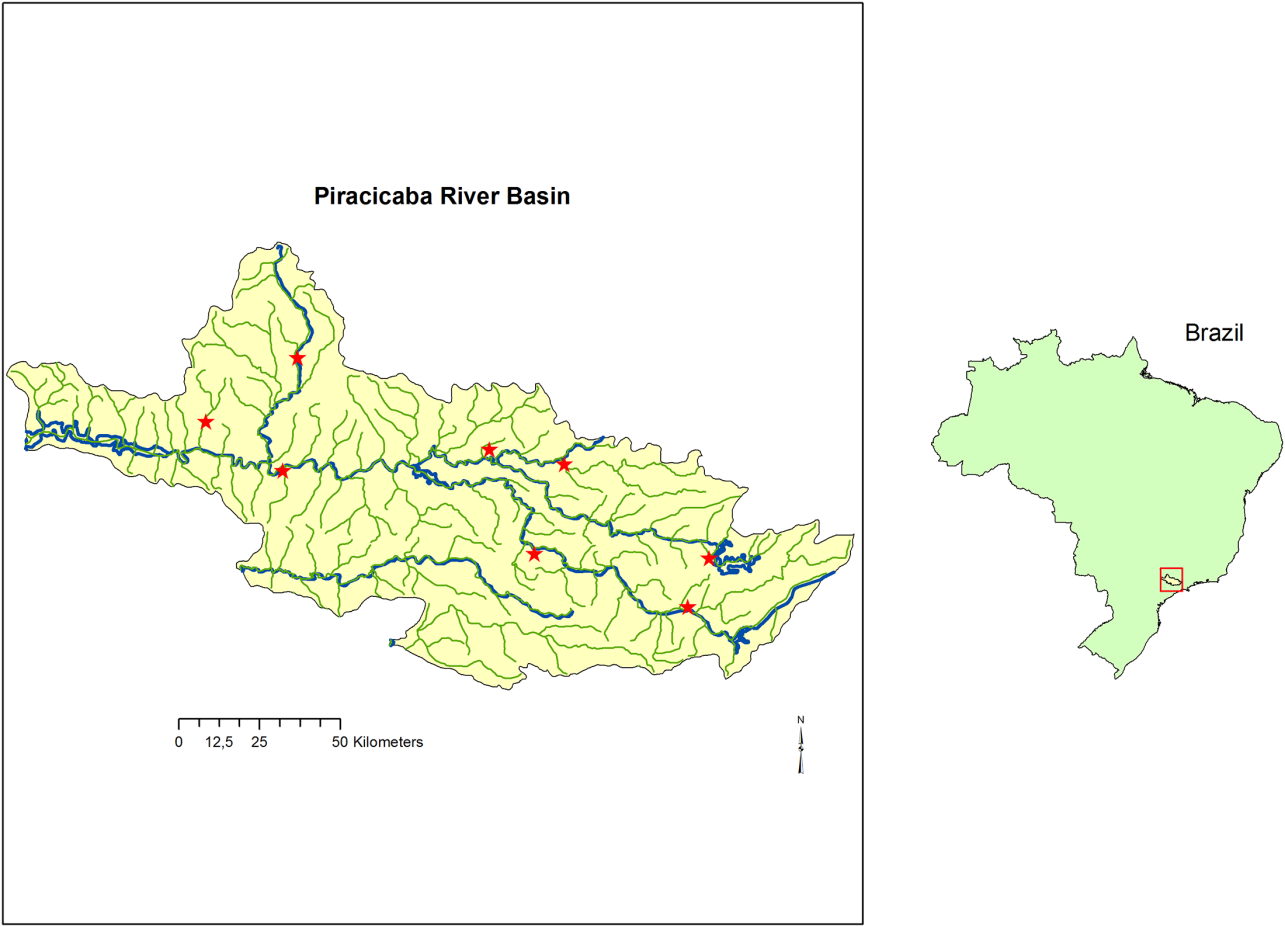
Map of Piracicaba River Basin, São Paulo, Brazil, including hydrology, main rivers and eight points of blood sample collection of 504 equines (red stars), 2012–2013.

Most cases of BSF are concentrated in the Southeast region of Brazil with sparse cases in other states, especially in the South. Between 2007 and 2015, 512 cases were confirmed in the state of São Paulo, 80% (409 cases) in 31 of 90 municipalities in the study region (around 5 million inhabitants) (data from Epidemiological Surveillance Information System, São Paulo, 2016). The fatality rate in São Paulo is still very high compared to other regions; in 2010 and 2011 rates reached 48.6% and 47.3% respectively (São Paulo, 2013). Most BSF case reports in the state of São Paulo were related to the sites of infection (LSI) in the Piracicaba River Basin, which is classified as a BSF risk region in the state [8] (Fig 1).

Due to their propensity to support high density populations of *A*. *sculptum* [9], horses have been proposed to be a reliable sentinel animal for BSF. However, better data is needed to support the use of the seroprevalence surveillance of horses in estimating the risk of human BSF transmission in unknown occurrence areas. The aim of this research is to study the seroprevalence of antibodies to *R*. *rickettsii*, *R*. *bellii* and *R*. *parkeri* in equines in endemic and non-endemic areas of the Piracicaba River Basin, state of São Paulo and to compare the seroprevalence of anti-rickettsial antibodies with the incidence of human BSF cases in this region. Based on this data, we assess the potential use of this domestic species as a sentinel animal for detection of *R*. *rickettsii* distribution and a predictive model for BSF human transmission risk.

## Methods

A cross-sectional study with a serological survey in equines was performed to evaluate prevalence for three species of Rickettsia in eight areas in the Piracicaba River Basin, a region with the highest number of reported cases of BSF in São Paulo, during the years 2012–2013.

The sample size required was determined based on the estimation of the prevalence of Rickettsia infection in horses from endemic areas of São Paulo previously made by Horta et al. (2004) [10]. Based on the estimated prevalence of Rickettsia infection in horses of 77%, it was determined that a sample size of at least 57 animals in each location, would afford a 90% confidence level.

Four sites were chosen among the municipalities with the highest number of reported cases of BSF in the last 15 years based on the Epidemiological Surveillance System. Other four sites were selected in municipalities where there were no reported human cases in the last 15 years (1998 to 2013).

Among those municipalities with the highest reported rate of BSF infections, we have chosen the areas with most likely site of infection (LSI) in Piracicaba (41 cases), Jaguariuna (24 cases) cities alongside the river Jaguarí; Valinhos (38 cases) alongside the river Capivari; and Amparo (9 cases) located in the north region of the basin (data from Epidemiological Surveillance Information System, São Paulo, 2015). The eight research sites were distributed in such a way in order to include the main areas of the Piracicaba River Basin and the banks of its major rivers (Fig 1).

The sites in the non-endemic areas were chosen in riverbanks based on the presence of capybaras, areas of degraded field, both of which are areas known to have a higher occurrence of both vectors and hosts, potentially enabling human infection. The municipalities chosen were, Rio Claro and São Pedro, cities located alongside Corumbataí river in northwest of the basin, Bragança Paulista and Atibaia, cities located in the eastern and southern regions of the basin alongside Atibaia and Jaguarí rivers respectively (Fig 1).

After obtaining owner’s authorization, blood samples of equines were drawn from the jugular vein on the horses; after clot retraction samples were centrifuged at 3000 rpm for 10 minutes to obtain serum, thereafter were transferred and stored in polypropylene microcentrifuge tubes (Eppendorf^®^) at −20°C until use.

The equine serum samples were first tested for the presence of antibodies reacting against *R*. *rickettsii* by IFA, only the sera that yielded positive in this step were also tested for the presence of antibodies reacting with *R*. *bellii*, and *R*. *parkeri*, as these three *Rickettsia* species are the most common in the area of investigation [11] [12] [13]. While *R*. *rickettsii* is the etiologic agent of BSF and has been found in *A*. *sculptu*m [14], *R*.*bellii* and *R*. *parkeri* complex have also been found in *Amblyomma dubitatum* ticks in this area [15]. *A*. *dubitatum* species is often found coexisting with *A*. *scultpum* in southeastern Brazil. Therefore these three Rickettial species are expected to occur in the studied area, it is important to establish the seroprevalence for *R*. *bellii* and *R*. *parkeri*, in order to analyze the most likely homologous reaction among the antigens tested as described elsewhere [10].

Serum samples were afterwards thawed and processed in laboratory by indirect immunofluorescence assay (IFA). IFA slides were produced using three rickettsial antigens isolated in Brazil: Taiaçu strain of *R*. *rickettsia*, Mogi strain of *R*. *bellii* [11], and *R*. *parkeri* AT24 strain A [12]. The antigens were fixed on slides in accordance with Horta et al. (2004) [10].

To perform the IFA an aliquot of serum was diluted in PBS (phosphate buffered saline solution) and incubated at 37°C for 30 minutes on a slide containing Rickettsia antigen as well as positive and negative controls consisting of wells incubated with a known reactive serum and others incubated with a naïve serum, respectively. The slides were then washed in PBS and dried. Fluorescein isothiocyanate (FITC) labeled sheep anti-horse IgG conjugate (Sigma) was applied to each well, and incubated in a humid chamber at 37°C for 30 min.

Sera were diluted in twofold increments with PBS starting from 1:64 dilution. Endpoint titles against each Rickettsia species were determined by testing serial twofold serum dilutions. Negative and positive controls used on each slide were at a dilution of 1:64. Slides were washed two times with wash buffer added with Evans blue during 7 minutes and left protected from light until the reading (within 24 hours). The slides were read by two biologists using an immunofluorescence microscope.

A positive title was considered if a fluorescent signal could be detected using a dilution of the serum ≥ 1:64. A previously established positive control serum that yielded positive in IFA with titles above 1:1024 for *R*. *parkeri*, *R*. *bellii* and *R*. *rickettsii* was used. The negative control serum was obtained from a horse that yielded a negative result by IFA for each *R*. *parkeri*, *R*. *bellii* and *R*. *rickettsii*.

Sera showing a title at least four-fold higher for one of the tested species of Rickettsia than the others were considered homologous reaction to the species with the highest title [10].

The geometric mean titers were calculated for each municipality and grouped for endemic and non endemic areas for BSF. Seroprevalence from endemic and non endemic areas were compared using Kruskal-Wallis, a non-parametric test, with the level of significance of 5%.

The project was approved by the Ethics Committee in Research of Animals CEUA/UNICAMP, November-2011/2563-1. We took specific access permission from owners of the land and animals. The field study did not involve endangered or protected species.

## Results

The serological survey was performed in 504 horses, around 63 in each investigated area. Among the 504 equines 183 (36.3%) were seropositive (≥ 1:64) for *Rickettsia rickettsii* antigens. Among those, 110 (60.1%) were autochthonous animals from municipalities with notification of BSF in the last 15 years, and 73 (39.9%) were from areas without human disease notification. All animals tested were autochthonous to the study areas and had not circulated into other areas.

In the four endemic areas the seroprevalence for *Rickettsia rickettsii* immunoreactive antibodies was 44.5% (range: 38.2%–54.7%), and in locations without notification of BSF, the seroprevalence was 28.4% (range: 6.1%–53%) (Table 1). Noteworthy high *Rickettsia rickettsii* seropositivity was detected in equines that live in areas without reported human cases, especially in Bragança Paulista and Rio Claro, 53% and 43.5% respectively. A recent notification of one confirmed case of BSF by the Epidemiological Surveillance team in Rio Claro (November 2014) shows the existing risk and the high likelihood of human transmission in this municipality (data from Epidemiological Surveillance Information System, São Paulo, 2015). Geometric mean titles were different between groups of municipalities with and without human cases (p = 0.012) (Table 1).

**Table 1:** Seroprevalence (%) for *Rickettsia rickettsii* by indirect immunofluorescence assay and geometric mean titer in equines, Piracicaba River Basin, state of São Paulo, Brazil, 2012–2013.

Notification of BSF	Local of collection	Number of samples	GMT^a^ *R*. *rickettsii*	Seroprevalence^b^ (%) (n° reactive)
Yes	Jaguariúna	57	108.38	43.9 (25)
Yes	Amparo	58	114.04	41.4 (24)
Yes	Valinhos	68	167.11	38.2 (26)
Yes	Piracicaba	64	128.0	54.7 (35)
Subtotal		247	8.69	44.5 (110)
No	Rio Claro	62	112.58	43.5 (27)
No	Bragança	66	197.89	53 (35)
No	Atibaia	63	143.68	9.5 (6)
No	São Pedro	66	111.43	6.1 (5)
Subtotal		257	4.15	28.4 (73)
Total		504		36.3 (183)

It was obtained p value = 0.012 of Kruskal Wallis Test for independent groups (with and without human cases) with significance of 5%.

^a^ GMT—Geometric mean titers

^b^ Reactive sample for *R*. *rickettsii*, titer ≥ 1:64.

Among the 183 equines seropositive for *R*. *rickettsii*, we also detected reactivity for *R*. *bellii* in 86 (46.9%) and in 78 (42.6%) for *R*. *parkeri*. It was not possible to distinguish cross reactivity or exposition to *R*. *belli* and *R*. *parkeri*. Titers suggesting homologous sera were found in 20 equines for *R*. *rickettsii* in all sites off collection, four for *R*. *parkeri* in Piracicaba and two for *R*. *bellii* in Amparo and Piracicaba (Table 2, S1 File).

**Table 2:** Reciprocal antibody titers by indirect immunofluorescence assay for *Rickettsia* spp. in 504 equines, Piracicaba River Basin, São Paulo, Brazil, 2012–2013.

Municipalities	Human cases	*R*. *rickettsii*^a^	*R*.*parkeri*^a^	*R*.*bellii*^a^
Jaguariuna	Yes	512	64	negative
		256	negative	64
Amparo	Yes	128	negative	1024
		256	64	64
Piracicaba	Yes	512	64	negative
		512	64	negative
		256	1024	negative
		64	512	negative
		64	256	negative
		128	negative	512
		64	256	negative
Valinhos	Yes	256	64	64
		256	64	64
		256	64	64
		256	64	negative
		256	64	64
		256	negative	64
Rio Claro	Yes	256	64	64
Atibaia	No	256	negative	64
		256	64	64
Bragança	No	1024	64	64
		256	64	negative
		256	64	negative
		1024	64	64
		256	64	negative
São Pedro	No	256	64	64
Total homologous (n = 26)		20	4	2

^a^Data from S1 File.

To assess the presence of other potential hosts for *A*. *sculptum* we examined the environmental data and animals reported to be found in the sites investigated, particularly in areas of riparian forests, degraded and clean pastures, and on the banks of rivers and ponds (Table 3). Other potential reservoirs of BSF, including both domestic and wild animals were found.

**Table 3:** Environmental characteristics and presence of animals in areas with and without notification of BSF in Piracicaba River Basin, state of São Paulo, 2012–2013.

Notification of BSF	Municipalities	Rural/Urban	Type of vegetation	Capybara presence	Animals presence
Yes	Amparo^a^	Rural/Periurban	Riparian forest	Yes	Horses, Dogs, Wild animals
Yes	Jaguariúna^a^	Rural	Remnants forest, Orchard	Yes	Horses, Dogs, Wild animals
Yes	Piracicaba^a^	Rural	Dirty pasture, Remnants forest	Yes	Dogs, Wild animals
Yes	Valinhos^a^	Rural	Remnants forest, Clean pasture, Orchard	Yes	Wild animals
No	Rio Claro	Periurban	Riparian forest	Yes	Horses, Dogs, Wild animals
No	Bragança	Rural	Riparian forest	Yes	Wild animals
No	Atibaia	Rural	Remnants forest, Pond, Clean pasture	Yes	Horses, Dogs, Wild animals
N	São Pedro	Rural	Riparian forest	No	No

^a^The areas of equine blood collection coincide with likely sites of infection (LSI) in 4 municipalities with reported cases.

In almost all municipalities the presence of capybaras and wild animals were reported. Only in São Pedro there were no reports of existing capybaras or other potential host animals at the moment of investigation (Table 3).

## Discussion

We tested the seroprevalence for *R*. *rickettsii* in a large sample of equines (504) autochthonous to eight areas in the Piracicaba River Basin. Seropositivity of equines in all LSI indicates *R*. *rickettsii* exposure suggesting transmission of the pathogen in this domestic species. We note a high proportion of seropositivity to *R*. *rickettsii* in equines from BSF endemic, but also in those testedin non endemic regions, which had high prevalence in one municipality Bragança (53%). Reports of capybaras and other wild animals involved in the natural cycle of BSF in areas of high seroprevalence in equines indicate a strong potential for disease transmission in humans. This possibility is likely given that *A*. *sculptum* is a vector that feeds on both capybaras and equines which allows it to bridge the infection between these animals [16].

We were surprised by the findings of high seroprevalence for *R*. *rickettsii* in areas without reported human BSF cases within the last 15 years. The results presented here strongly suggest the potential risk for human transmission in these areas, as seen recently in Rio Claro by the first human case notified in November, 2014 [8].

Some blood samples reactive to *R*. *rickettsii* were also reactive to *R*. *bellii* (46.9%) and *R*. *parkeri* (42.6%) suggesting either cross-reactivity between these Rickettsia species or co-infection with different Rickettsia species. However, animal samples that yielded a homologous *R*. *rickettsia* sera confirmation showed that this species were transmitted among the tested animals. Although, it cannot be ruled out that other species of Rickettsia may be responsible for infection in equines, there is evidence of previous transmission of species of Rickettsia of the spotted fever group in equines in all sites. Horta et al. (2004) [10] also have showed that equines in the state of São Paulo infected with *R*. *rickettsii* generate antibodies that cross-react with *R*. *bellii* or *R*. *parkeri* and vice versa.

*R*. *belli* is a common agent infecting ticks in the areas of investigation and its presence has been confirmed by PCR in *A*. *sculptum* and *A*. *dubitatum* [13] whereas in the same study region, *R*. *parkeri* has been isolated from *A*. *dubitatum* [15]. Few cases (2) of cross-reaction between *R*. *bellii* and *R*. *rickettsii* were probably due to the species of vectors found in the study animals. *A*. *dubitatum*, specie often infected by *R*. *bellii* was found parasitizing horses in the region. Worth noting that immature stages of Amblyomma do not have food selectivity, thus parasitize several species of mammals. The prevalence of *R*. *bellii* infections is most likely underestimated in our sample since we tested for *R*. *bellii* only for samples that were tested positive for R. rickettsia.

Due to its availability and ease of use in routine laboratory settings, IFA is still the most important tool in the diagnosis and epidemiological investigation of rickettsial infections in humans and animals, [17]. However, in comparison with PCR, IFA is limited by its lack of specificity and lack of discrimination between different Rickettsia species, also by lack of timeframe with regards to the active infection. It takes time for the infected animal or patient to seroconvert to produce sufficient antibodies, and simply based on seropositivity we cannot estimate if the infection is ongoing or simply that the animal has been exposed at some point in time to *Rickettsia* sp.

Studies have shown high prevalence of antibody to *R*. *rickettsii* in horses, particularly in endemic areas of BSF. Horta et al. (2004) [10] estimated seroprevalence of 77.3% in tested horses in a rural endemic area in Pedreira, state of São Paulo. Sangione et al. (2005) also conducted a serological survey in three endemic areas (Pedreira) and in three non-endemic (Cotia, Pirassununga and Porto Feliz) in São Paulo. These data also showed seropositivity to *R*. *rickettsii* in horses in areas where human cases were reported, while there were no immunoreactive sera in horses from non-endemic areas, thereby reinforcing the predictive potential of using horses as sentinel animals of human cases of BSF.

Statistical differences were observed in seropositivity to *R*. *rickettsii* between areas with and without human cases were also found in our study (p = 0.012). Although Atibaia and São Pedro have shown low prevalence of seropositivity (6.1% and 9.5%) respectively, Rio Claro (one case only recently) and Bragança Paulista showed values as high as known in endemic areas. It is worth noting the absence of capybaras in Sao Pedro, area with the lowest seroprevalence in horses. We found seropositivity of equines in both areas but higher in areas with human cases. The results reinforce that serology in horses may be a useful tool in surveillance of BSF, and could be incorporated in surveillance routine.

Previous studies have examined the seropositivity in horses in BSF non endemic areas. Among these studies, Moraes–Filho et al. (2009) [18] found a low incidence of reactive animals (17.6%), but the provenance of these animals was unknown, and most of them were stray animals, different from the present study in which horses were autochthonous. In Caratinga in the state of Minas Gerais, Cardoso et al. (2006) [19] found reactive serology in 3 of 18 (17%) horses’ samples and detected DNA of pathogenic Rickettsia in arthropod vectors in a region with no reported cases of BSF in the last 12 years. In Paraná state, Batista et al. (2010) [20] also found horses and dogs positive for *R*. *rickettsii* in areas with no reported human case.

In São José dos Pinhais, state of Paraná, where the first report of BSF case in the state took place, Freitas et al. (2010) [7] reported 7 (9.3%) reactive samples for *R*. *rickettsii* of 75 cart horses. It was hypothesized that the transmission of Rickettsia in this region particularly among cart horses can present a serious concern for disease spread and human exposure because they can travel great distances, dispersing infected ticks.

There are some possible explanations for the absence of human cases in areas with seropositive horses: the short time exposure to ticks in humans which usually withdraw the tick from the body before it can transmit the bacteria through saliva; and also there are different ways and intensity of exposure to infected horses, ranging from riding for work and/or leisure activities, and more infrequent and occasional contacts with work horses. These different behaviors may also influence the risk of disease [10]. Our data suggests that an enzootic cycle of Rickettsia infections is already established in the Piracicaba River Basin, although a little ricketsial infection rate in ticks may hinder human infection. On the other hand, surveillance of human cases of BSF may be insufficient to predict an imminent risk of human transmission. Moreover underreporting of human cases as well as missed diagnoses of the disease in areas where there are no reported cases of BSF may occur and explain the apparent absence of disease in humans despite the proven circulation of *R*. *rickettsii*, as evidenced in horses in this study.

Areas with detection of *R*. *rickettsii* in sentinel animals, presence of hosts as capybara but no human cases may need health staff training to increase awareness and accurate diagnosis of BSF, and improve disease and acarology surveillance. However, epidemiological surveillance in the municipalities studied is hampered by low availability of resources and inadequate training of the health services teams.

Investigation of only eight points in the basin does not allow depth evaluation of the environmental patterns involved in the circulation of Rickettsia, however the large sample size of equines tested and their distribution into distinct parts of the Piracicaba River Basin increase confidence in the findings of this study. Other studies with larger coverage area and collection of environmental data could facilitate the understanding causes of human transmission in some regions and not in others in the last decades. Based on our large sample size, we believe our data on the seroprevalence of *R*. *rickettsii* in autochthonous equines raises an alarm of the high likelihood of impending human transmission of BSF in areas without known previous cases but where there are ecological conditions of transmission of Rickettsia of the spotted fever group.

## Supporting Information

S1 File Individual data from equines. (PDF)
