# Supplementary material for: High Seroprevalence for Rickettsia rickettsii in Equines Suggests Risk of Human Infection in Silent Areas for the Brazilian Spotted Fever
Source: PLoS One. 2016 Apr 11;11(4):e0153303. doi: 10.1371/journal.pone.0153303 (PMC4827800; doi:10.1371/journal.pone.0153303)
Supplement: S1 File — (PDF) [file pone.0153303.s001.pdf]

long	lat	município	month	ano coleta	sample	age	sex	RIFI	R. rickettsi	R. belli	R. Parkeri
299264	7465072	Jaguariuna	10	2012	1	7	m	reagente	1/512	0	1\64
299264	7465072	Jaguariuna	10	2012	2	9	f	não reagente	0	0	0
299264	7465072	Jaguariuna	10	2012	3	7	m	não reagente	0	0	0
299264	7465072	Jaguariuna	10	2012	4	6	m	não reagente	0	0	0
299264	7465072	Jaguariuna	10	2012	5	7	m	não reagente	0	0	0
299264	7465072	Jaguariuna	10	2012	6	5	m	não reagente	0	0	0
299264	7465072	Jaguariuna	10	2012	7	3	m	não reagente	0	0	0
299264	7465072	Jaguariuna	10	2012	8	2	m	reagente	1\64	1/128	0
299264	7465072	Jaguariuna	10	2012	9	13	m	não reagente	0	0	0
299264	7465072	Jaguariuna	10	2012	10	5	m	não reagente	0	0	0
299264	7465072	Jaguariuna	10	2012	11	12	f	reagente	1/256	1\64	0
299264	7465072	Jaguariuna	10	2012	12	6	f	reagente	1\64	1\128	1\64
299264	7465072	Jaguariuna	10	2012	13	15	f	não reagente	0	0	0
299264	7465072	Jaguariuna	10	2012	14	12	m	reagente	1\64	1/128	1\64
299264	7465072	Jaguariuna	10	2012	15	15	m	reagente	1\64	1/128	1\64
299264	7465072	Jaguariuna	10	2012	16	6	m	não reagente	0	0	0
299264	7465072	Jaguariuna	10	2012	17	2	m	não reagente	0	0	0
299264	7465072	Jaguariuna	10	2012	18	8	m	reagente	1/128	1/256	0
299264	7465072	Jaguariuna	10	2012	19	3	f	não reagente	0	0	0
299264	7465072	Jaguariuna	10	2012	20	4	m	não reagente	0	0	0
310002	7487158	Amparo	6	2013	21	10	m	reagente	1/128	1/256	1\64
310002	7487158	Amparo	6	2013	22	12	m	reagente	1/128	1/128	1\64
310002	7487158	Amparo	6	2013	23	2	m	não reagente	0	0	0
310002	7487158	Amparo	6	2013	24	13	m	não reagente	0	0	0
310002	7487158	Amparo	6	2013	25	18	f	reagente	1/256	0	0
310002	7487158	Amparo	6	2013	26	10	f	reagente	1/128	0	0
310002	7487158	Amparo	6	2013	27	10	m	não reagente	0	0	0
310002	7487158	Amparo	6	2013	28	7	m	reagente	1/128	1/1024	0
310002	7487158	Amparo	6	2013	29	8	m	não reagente	0	0	0
310002	7487158	Amparo	6	2013	30	12	m	reagente	1/256	1/512	0

306050	7490432	Amparo	6	2013	31	3	m	não reagente	0	0	0
306050	7490432	Amparo	6	2013	32	3	m	não reagente	0	0	0
306050	7490432	Amparo	6	2013	33	3	m	reagente	1/128	1/128	1\64
306050	7490432	Amparo	6	2013	34	3	m	reagente	1\64	1/128	1\64
306050	7490432	Amparo	6	2013	35	3	m	reagente	1/256	1/256	1/256
314188	7481667	Amparo	6	2013	36	18	m	não reagente	0	0	0
314188	7481667	Amparo	6	2013	37	10	f	não reagente	0	0	0
314188	7481667	Amparo	6	2013	38	25	m	reagente	1/256	1/256	0
314188	7481667	Amparo	6	2013	39	4	m	não reagente	0	0	0
311326	7485971	Amparo	6	2013	40	7	m	não reagente	0	0	0
311326	7485971	Amparo	6	2013	41	7	f	reagente	1\64	1/256	0
311326	7485971	Amparo	6	2013	42	1	m	não reagente	0	0	0
311597	7485564	Amparo	6	2013	43	5	m	não reagente	0	0	0
311597	7485564	Amparo	6	2013	44	1	m	não reagente	0	0	0
314466	7485157	Amparo	6	2013	45	12	m	reagente	1\64	1\64	1\64
314466	7485157	Amparo	6	2013	46	11	m	reagente	1\64	1/128	1\64
314466	7485157	Amparo	6	2013	47	9	f	reagente	1/256	1\64	1\64
314466	7485157	Amparo	6	2013	48	28	m	não reagente	0	0	0
314466	7485157	Amparo	6	2013	49	15	m	não reagente	0	0	0
314466	7485157	Amparo	6	2013	50	14	f	reagente	1\64	0	1\64
314466	7485157	Amparo	6	2013	51	4	m	não reagente	0	0	0
314466	7485157	Amparo	6	2013	52	16	m	reagente	1\64	0	1\64
314466	7485157	Amparo	6	2013	53	5	m	reagente	1\64	0	0
314466	7485157	Amparo	6	2013	54	13	m	reagente	1/256	0	0
314466	7485157	Amparo	6	2013	55	17	f	reagente	1\64	1\64	0
308775	7486981	Amparo	6	2013	56	9	m	não reagente	0	0	0
308775	7486981	Amparo	6	2013	57	3	m	reagente	1/128	1\64	1\64
308775	7486981	Amparo	6	2013	58	12	m	não reagente	0	0	0
308775	7486981	Amparo	6	2013	59	3	m	não reagente	0	0	0
308775	7486981	Amparo	6	2013	60	3	m	não reagente	0	0	0
308775	7486981	Amparo	6	2013	61	13	m	reagente	1/256	0	0

308775	7486981	Amparo	6	2013	62	2	m	não reagente	0	0	0
308775	7486981	Amparo	6	2013	63	2	f	reagente	1\64	1\64	1\64
308775	7486981	Amparo	6	2013	64	4	f	não reagente	0	0	0
308775	7486981	Amparo	6	2013	65	18	m	não reagente	0	0	0
308775	7486981	Amparo	6	2013	66	4	m	não reagente	0	0	0
308775	7486981	Amparo	6	2013	67	2	m	não reagente	0	0	0
308775	7486981	Amparo	6	2013	68	9	f	não reagente	0	0	0
308775	7486981	Amparo	6	2013	69	13	m	reagente	1\64	1/512	0
308775	7486981	Amparo	6	2013	70	15	m	não reagente	0	0	0
308775	7486981	Amparo	6	2013	71	20	m	não reagente	0	0	0
308775	7486981	Amparo	6	2013	72	15	f	reagente	1\64	1\64	1\64
308775	7486981	Amparo	6	2013	73	16	m	não reagente	0	0	0
308775	7486981	Amparo	6	2013	74	5	m	não reagente	0	0	0
308775	7486981	Amparo	6	2013	75	5	f	não reagente	0	0	0
308775	7486981	Amparo	6	2013	76	5	f	não reagente	0	0	0
308775	7486981	Amparo	6	2013	77	4	f	não reagente	0	0	0
308775	7486981	Amparo	6	2013	78	6	f	não reagente	0	0	0
239018	7526191	Rio Claro	6	2013	79	4	m	não reagente	0	0	0
239018	7526191	Rio Claro	6	2013	80	5	f	reagente	1/256	0	1\64
239018	7526191	Rio Claro	6	2013	81	4	f	não reagente	0	0	0
239018	7526191	Rio Claro	6	2013	82	24	m	reagente	1\64	0	0
239018	7526191	Rio Claro	6	2013	83	8	f	reagente	1/128	1\64	0
239018	7526191	Rio Claro	6	2013	84	7	m	reagente	1/256	0	0
239018	7526191	Rio Claro	6	2013	85	5	m	não reagente	0	0	0
239018	7526191	Rio Claro	6	2013	86	11	m	reagente	1/128	0	0
239018	7526191	Rio Claro	6	2013	87	2	f	não reagente	0	0	0
239018	7526191	Rio Claro	6	2013	88	3	m	reagente	1\64	1/512	0
239018	7526191	Rio Claro	6	2013	89	7	m	reagente	1/256	1\64	1\64
239018	7526191	Rio Claro	6	2013	90	4	f	reagente	1/256	1/128	1\64
239018	7526191	Rio Claro	6	2013	91	12	m	reagente	1\64	1\64	1\64
239018	7526191	Rio Claro	6	2013	92	7	f	não reagente	0	0	0

239018	7526191	Rio Claro	6	2013	93	8	f	não reagente	0	0	0
237613	7519546	Rio Claro	6	2013	94	18	m	não reagente	0	0	0
237613	7519546	Rio Claro	6	2013	95	12	m	não reagente	0	0	0
237613	7519546	Rio Claro	6	2013	96	15	m	não reagente	0	0	0
237613	7519546	Rio Claro	6	2013	97	15	m	não reagente	0	0	0
237613	7519546	Rio Claro	6	2013	98	12	f	não reagente	0	0	0
237613	7519546	Rio Claro	6	2013	99	25	m	não reagente	0	0	0
237613	7519546	Rio Claro	6	2013	100	15	m	reagente	1/128	0	0
237613	7519546	Rio Claro	6	2013	101	15	f	não reagente	0	0	0
237613	7519546	Rio Claro	6	2013	102	8	m	reagente	1/128	0	0
237613	7519546	Rio Claro	6	2013	103	8	m	não reagente	0	0	0
237613	7519546	Rio Claro	6	2013	104	10	m	reagente	1/256	0	0
237613	7519546	Rio Claro	6	2013	105	7	m	reagente	1\64	0	0
237613	7519546	Rio Claro	6	2013	106	18	f	não reagente	0	0	0
237613	7519546	Rio Claro	6	2013	107	10	m	reagente	1\64	0	1\64
237613	7519546	Rio Claro	6	2013	108	7	f	reagente	1\64	1/128	1\64
237613	7519546	Rio Claro	6	2013	109	6	m	reagente	1\64	0	0
237613	7519546	Rio Claro	6	2013	110	20	f	não reagente	0	0	0
237613	7519546	Rio Claro	6	2013	111	18	f	reagente	1/256	0	0
237613	7519546	Rio Claro	6	2013	112	18	m	reagente	1/128	0	0
237613	7519546	Rio Claro	6	2013	113	7	m	reagente	1\64	1\64	0
234776	7525954	Rio Claro	6	2013	114	5	m	não reagente	0	0	0
234776	7525954	Rio Claro	6	2013	115	6	m	não reagente	0	0	0
234776	7525954	Rio Claro	6	2013	116	10	f	reagente	1/128	0	0
234776	7525954	Rio Claro	6	2013	117	3	m	não reagente	0	0	0
234776	7525954	Rio Claro	6	2013	118	3	m	não reagente	0	0	0
234776	7525954	Rio Claro	6	2013	119	3	m	não reagente	0	0	0
234776	7525954	Rio Claro	6	2013	120	7	m	não reagente	0	0	0
234776	7525954	Rio Claro	6	2013	121	3	m	não reagente	0	0	0
234776	7525954	Rio Claro	6	2013	122	5	m	não reagente	0	0	0
234776	7525954	Rio Claro	6	2013	123	6	m	não reagente	0	0	0

234776	7525954	Rio Claro	6	2013	124	3	f	não reagente		0	0	0
234776	7525954	Rio Claro	6	2013	125	5	m	não reagente		0	0	0
234776	7525954	Rio Claro	6	2013	126	5	m	não reagente		0	0	0
234776	7525954	Rio Claro	6	2013	127	8	m	não reagente		0	0	0
234776	7525954	Rio Claro	6	2013	128	7	m	reagente	1\64	1\64	1\64	
234776	7525954	Rio Claro	6	2013	129	2	f	não reagente		0	0	0
234776	7525954	Rio Claro	6	2013	130	8	f	não reagente		0	0	0
227067	7527609	Rio Claro	6	2013	131	9	m	não reagente		0	0	0
227067	7527609	Rio Claro	6	2013	132	6	m	reagente	1/256		0	1/128
227067	7527609	Rio Claro	6	2013	133	5	f	reagente	1\64		0	1\64
227067	7527609	Rio Claro	6	2013	134	4	f	reagente	1\64		0	0
227067	7527609	Rio Claro	6	2013	135	3	f	não reagente		0	0	0
227067	7527609	Rio Claro	6	2013	136	3	f	reagente	1\64		0	0
227067	7527609	Rio Claro	6	2013	137	16	f	não reagente		0	0	0
227067	7527609	Rio Claro	6	2013	138	4	m	não reagente		0	0	0
227067	7527609	Rio Claro	6	2013	139	3	f	reagente	1\64		0	1\64
227067	7527609	Rio Claro	6	2013	140	9	m	reagente	1/256		0	0
198451	7505202	São Pedro	6	2013	141	10	f	não reagente		0	0	0
198451	7505202	São Pedro	6	2013	142	10	f	não reagente		0	0	0
198451	7505202	São Pedro	6	2013	143	25	m	não reagente		0	0	0
198451	7505202	São Pedro	6	2013	144	25	m	não reagente		0	0	0
198451	7505202	São Pedro	6	2013	145	12	m	não reagente		0	0	0
198451	7505202	São Pedro	6	2013	146	12	f	não reagente		0	0	0
198451	7505202	São Pedro	6	2013	147	12	f	não reagente		0	0	0
198451	7505202	São Pedro	6	2013	148	14	f	não reagente		0	0	0
198304	7507289	São Pedro	6	2013	149	5	f	não reagente		0	0	0
198304	7507289	São Pedro	6	2013	150	3	f	não reagente		0	0	0
198304	7507289	São Pedro	6	2013	151	7	f	não reagente		0	0	0
198304	7507289	São Pedro	6	2013	152	12	f	não reagente		0	0	0
197758	7502315	São Pedro	6	2013	153	3	m	não reagente		0	0	0
197758	7502315	São Pedro	6	2013	154	24	m	não reagente		0	0	0

197758	7502315	São Pedro	6	2013	155	8	m	não reagente	0	0	0
197758	7502315	São Pedro	6	2013	156	8	m	não reagente	0	0	0
197758	7502315	São Pedro	6	2013	157	7	f	não reagente	0	0	0
197758	7502315	São Pedro	6	2013	158	26	f	reagente	1/256	1\64	1\64
197758	7502315	São Pedro	6	2013	159	10	m	não reagente	0	0	0
197758	7502315	São Pedro	6	2013	160	11	f	não reagente	0	0	0
197758	7502315	São Pedro	6	2013	161	23	m	não reagente	0	0	0
197758	7502315	São Pedro	6	2013	162	7	m	não reagente	0	0	0
197758	7502315	São Pedro	6	2013	163	14	m	não reagente	0	0	0
197758	7502315	São Pedro	6	2013	164	9	m	não reagente	0	0	0
197758	7502315	São Pedro	6	2013	165	8	m	não reagente	0	0	0
197758	7502315	São Pedro	6	2013	166	9	m	não reagente	0	0	0
197758	7502315	São Pedro	6	2013	167	8	m	não reagente	0	0	0
197758	7502315	São Pedro	6	2013	168	10	m	não reagente	0	0	0
197758	7502315	São Pedro	6	2013	169	4	m	não reagente	0	0	0
197758	7502315	São Pedro	6	2013	170	23	m	não reagente	0	0	0
197758	7502315	São Pedro	6	2013	171	15	m	não reagente	0	0	0
197758	7502315	São Pedro	6	2013	172	7	m	não reagente	0	0	0
197758	7502315	São Pedro	6	2013	173	15	m	não reagente	0	0	0
197758	7502315	São Pedro	6	2013	174	23	f	não reagente	0	0	0
197758	7502315	São Pedro	6	2013	175	13	m	não reagente	0	0	0
197758	7502315	São Pedro	6	2013	176	9	f	reagente	1\64	1/128	0
197758	7502315	São Pedro	6	2013	177	27	m	não reagente	0	0	0
197758	7502315	São Pedro	6	2013	178	15	f	não reagente	0	0	0
197758	7502315	São Pedro	6	2013	179	9	m	reagente	1\128	1\64	0
197758	7502315	São Pedro	6	2013	180	2	m	não reagente	0	0	0
197758	7502315	São Pedro	6	2013	181	12	m	não reagente	0	0	0
197758	7502315	São Pedro	6	2013	182	11	m	não reagente	0	0	0
197758	7502315	São Pedro	6	2013	183	8	f	não reagente	0	0	0
198373	7498257	São Pedro	5	2013	184	17	f	não reagente	0	0	0
198373	7498257	São Pedro	5	2013	185	13	m	não reagente	0	0	0

198373	7498257	São Pedro	5	2013	186	16	f	não reagente		0	0	0
198373	7498257	São Pedro	5	2013	187	17	f	não reagente		0	0	0
198373	7498257	São Pedro	5	2013	188	9	f	não reagente		0	0	0
198373	7498257	São Pedro	5	2013	189	13	m	não reagente		0	0	0
198373	7498257	São Pedro	5	2013	190	4	m	não reagente		0	0	0
198373	7498257	São Pedro	5	2013	191	18	m	reagente	1\64	1\64	1\64	
198373	7498257	São Pedro	5	2013	192	9	m	não reagente		0	0	0
198373	7498257	São Pedro	5	2013	193	11	m	não reagente		0	0	0
198373	7498257	São Pedro	5	2013	194	14	m	não reagente		0	0	0
198373	7498257	São Pedro	5	2013	195	7	f	não reagente		0	0	0
198373	7498257	São Pedro	5	2013	196	7	f	não reagente		0	0	0
198373	7498257	São Pedro	5	2013	197	14	m	não reagente		0	0	0
198373	7498257	São Pedro	5	2013	198	6	f	não reagente		0	0	0
198373	7498257	São Pedro	5	2013	199	15	m	não reagente		0	0	0
198373	7498257	São Pedro	5	2013	200	9	f	não reagente	1\128	1\64		0
198373	7498257	São Pedro	5	2013	201	15	m	não reagente		0	0	0
198373	7498257	São Pedro	5	2013	202	12	m	não reagente		0	0	0
198373	7498257	São Pedro	5	2013	203	10	m	não reagente		0	0	0
198373	7498257	São Pedro	5	2013	204	8	m	não reagente		0	0	0
198373	7498257	São Pedro	5	2013	205	16	f	não reagente		0	0	0
198373	7498257	São Pedro	5	2013	206	10	f	não reagente		0	0	0
298051	7486194	Jaguariuna	7	2013	207	4	m	não reagente		0	0	0
298051	7486194	Jaguariuna	7	2013	208	3	m	não reagente		0	0	0
298051	7486194	Jaguariuna	7	2013	209	4	m	não reagente		0	0	0
298051	7486194	Jaguariuna	7	2013	210	5	f	reagente	1\128	1/128		0
298051	7486194	Jaguariuna	7	2013	211	2	m	não reagente		0	0	0
298051	7486194	Jaguariuna	7	2013	212	2	m	reagente	1\64		0	0
298051	7486194	Jaguariuna	7	2013	213	3	f	reagente	1\64	1\64		0
298051	7486194	Jaguariuna	7	2013	214	3	f	reagente	1\64		0	0
296073	7487190	Jaguariuna	7	2013	215	2	f	reagente	1\64	1\64	1\64	
296073	7487190	Jaguariuna	7	2013	216	4	f	não reagente		0	0	0

296073	7487190	Jaguariuna	7	2013	217	3	m	não reagente	0	0	0
296073	7487190	Jaguariuna	7	2013	218	2	m	reagente	1\128	1/128	1\64
296073	7487190	Jaguariuna	7	2013	219	3	f	reagente	1\256	1\64	1/128
296073	7487190	Jaguariuna	7	2013	220	3	m	não reagente	0	0	0
296073	7487190	Jaguariuna	7	2013	221	3	m	reagente	1\64	1\64	0
296073	7487190	Jaguariuna	7	2013	222	4	m	não reagente	0	0	0
296073	7487190	Jaguariuna	7	2013	223	6	f	não reagente	0	0	0
302209	7461028	Valinhos		2012	224	8	f	reagente	128	64	64
302209	7461028	Valinhos		2012	225	6	f	reagente	256	64	64
302209	7461028	Valinhos		2012	226	6	m	reagente	256	0	0
302209	7461028	Valinhos		2012	227	6	m	não reagente	0	0	0
302209	7461028	Valinhos		2012	228	12	m	não reagente	0	0	0
302209	7461028	Valinhos		2012	229	5	m	reagente	64	0	0
302209	7461028	Valinhos		2012	230	8	m	reagente	256	64	64
302209	7461028	Valinhos		2012	231	6	m	não reagente	0	0	0
302209	7461028	Valinhos		2012	232	3	m	não reagente	0	0	0
302209	7461028	Valinhos		2012	233	8	m	não reagente	0	0	0
302209	7461028	Valinhos		2012	234	7	f	reagente	256	64	64
302209	7461028	Valinhos		2012	235	13	f	reagente	256	64	512
302209	7461028	Valinhos		2012	236	3	m	reagente	64	0	0
302209	7461028	Valinhos		2012	237	9	f	não reagente	0	0	0
302209	7461028	Valinhos		2012	238	10	m	reagente	256	64	0
302209	7461028	Valinhos		2012	239	17	f	reagente	256	0	0
302209	7461028	Valinhos		2012	240	12	m	reagente	64	64	128
302209	7461028	Valinhos		2012	241	28	m	reagente	256	64	64
302209	7461028	Valinhos		2012	242	12	f	reagente	256	64	256
302209	7461028	Valinhos		2012	243	11	f	reagente	256	64	128
302209	7461028	Valinhos		2012	244	8	m	não reagente	0	0	0
302209	7461028	Valinhos		2012	245	10	f	não reagente	0	0	0
302209	7461028	Valinhos		2012	246	20	m	reagente	256	64	64
302209	7461028	Valinhos		2012	247	17	m	não reagente	0	0	0

302209	7461028	Valinhos	2012	248	14	m	reagente	128	64	64
302209	7461028	Valinhos	2012	249	12	m	não reagente	0	0	0
302209	7461028	Valinhos	2012	250	15	m	não reagente	0	0	0
302209	7461028	Valinhos	2012	251	18	m	não reagente	0	0	0
302209	7461028	Valinhos	2012	252	10	m	não reagente	0	0	0
302209	7461028	Valinhos	2012	253	15	f	reagente	128	128	64
302209	7461028	Valinhos	2012	254	18	f	não reagente	0	0	0
302209	7461028	Valinhos	2012	255	12	m	não reagente	0	0	0
302209	7461028	Valinhos	2012	256	8	m	não reagente	0	0	0
302209	7461028	Valinhos	2012	257	6	f	não reagente	0	0	0
302209	7461028	Valinhos	2012	258	18	m	não reagente	0	0	0
302209	7461028	Valinhos	2012	259	10	m	não reagente	0	0	0
302209	7461028	Valinhos	2012	260	23	f	reagente	128	64	64
302209	7461028	Valinhos	2012	261	16	m	reagente	256	64	64
302209	7461028	Valinhos	2012	262	8	m	não reagente	0	0	0
302209	7461028	Valinhos	2012	263	20	m	não reagente	0	0	0
302209	7461028	Valinhos	2012	264	4	f	não reagente	0	0	0
302209	7461028	Valinhos	2012	265	18	f	reagente	256	128	512
302209	7461028	Valinhos	2012	266	13	m	reagente	256	64	64
302209	7461028	Valinhos	2012	267	23	m	reagente	128	64	256
302209	7461028	Valinhos	2012	268	17	m	reagente	64	0	0
302209	7461028	Valinhos	2012	269	20	m	reagente	256	64	0
300292	7457988	Valinhos	2013	270	13	m	não reagente	0	0	0
300292	7457988	Valinhos	2013	271	10	m	não reagente	0	0	0
300292	7457988	Valinhos	2013	272	5	f	não reagente	0	0	0
300292	7457988	Valinhos	2013	273	8	m	não reagente	0	0	0
300292	7457988	Valinhos	2013	274	14	m	reagente	1\64	1/128	0
300292	7457988	Valinhos	2013	275	14	m	reagente	1\128	1/128	1\64
300292	7457988	Valinhos	2013	276	10	m	não reagente	0	0	0
300292	7457988	Valinhos	2013	277	9	m	não reagente	0	0	0
300292	7457988	Valinhos	2013	278	4	m	não reagente	0	0	0

300292	7457988	Valinhos		2013	279	15	m	não reagente	0	0	0
300292	7457988	Valinhos		2013	280	7	m	não reagente	0	0	0
300292	7457988	Valinhos		2013	281	5	f	não reagente	0	0	0
300292	7457988	Valinhos		2013	282	4	m	não reagente	0	0	0
300292	7457988	Valinhos		2013	283	5	m	não reagente	0	0	0
300292	7457988	Valinhos		2013	284	9	m	não reagente	0	0	0
300292	7457988	Valinhos		2013	285	5	m	não reagente	0	0	0
300292	7457988	Valinhos		2013	286	10	m	não reagente	0	0	0
300292	7457988	Valinhos		2013	287	17	f	não reagente	0	0	0
300292	7457988	Valinhos		2013	288	17	m	não reagente	0	0	0
300292	7457988	Valinhos		2013	289	20	m	não reagente	0	0	0
300292	7457988	Valinhos		2013	290	20	m	não reagente	0	0	0
300292	7457988	Valinhos		2013	291	1 1/2	m	não reagente	0	0	0
343466	7444489	Atibaia	9	2013	292	6	f	não reagente	0	0	0
343466	7444489	Atibaia	9	2013	293	7	f	não reagente	0	0	0
343466	7444489	Atibaia	9	2013	294	8	f	não reagente	0	0	0
343466	7444489	Atibaia	9	2013	295	9	m	não reagente	0	0	0
343466	7444489	Atibaia	9	2013	296	12	m	não reagente	0	0	0
343466	7444489	Atibaia	9	2013	297	5	f	não reagente	0	0	0
343466	7444489	Atibaia	9	2013	298	15	m	não reagente	0	0	0
343466	7444489	Atibaia	9	2013	299	13	f	reagente	1\128	1\64	0
343466	7444489	Atibaia	9	2013	300	9	m	não reagente	0	0	0
343466	7444489	Atibaia	9	2013	301	5	m	não reagente	0	0	0
343466	7444489	Atibaia	9	2013	302	6	m	não reagente	0	0	0
343906	744713	Atibaia	9	2013	303	15	m	não reagente	0	0	0
343906	744713	Atibaia	9	2013	304	9	m	não reagente	0	0	0
343906	744713	Atibaia	9	2013	305	4	m	não reagente	0	0	0
343906	744713	Atibaia	9	2013	306	10	m	reagente	1\256	1\64	0
343906	744713	Atibaia	9	2013	307	5	m	reagente	1\256	1\64	1\64
343906	744713	Atibaia	9	2013	308	14	m	não reagente	0	0	0
343906	744713	Atibaia	9	2013	309	6	m	não reagente	0	0	0

343906	744713	Atibaia	9	2013	310	13	m	reagente	1\128	1/128		0
343906	744713	Atibaia	9	2013	311	6	f	não reagente		0	0	0
343906	744713	Atibaia	9	2013	312	3	m	não reagente		0	0	0
345952	744747	Atibaia	9	2013	313	3	m	não reagente		0	0	0
345952	744747	Atibaia	9	2013	314	4	f	não reagente		0	0	0
345952	744747	Atibaia	9	2013	315	9	m	não reagente		0	0	0
345952	744747	Atibaia	9	2013	316	2	f	não reagente		0	0	0
345952	744747	Atibaia	9	2013	317	5	f	não reagente		0	0	0
345952	744747	Atibaia	9	2013	318	5	m	reagente	1\128	1\64	1\64	
345952	744747	Atibaia	9	2013	319	15	m	não reagente		0	0	0
345952	744747	Atibaia	9	2013	320	19	m	não reagente		0	0	0
345952	744747	Atibaia	9	2013	321	4	m	não reagente		0	0	0
345952	744747	Atibaia	9	2013	322	3	m	não reagente		0	0	0
345952	744747	Atibaia	9	2013	323	13	m	não reagente		0	0	0
345952	744747	Atibaia	9	2013	324	5	m	não reagente		0	0	0
345952	744747	Atibaia	9	2013	325	9	m	não reagente		0	0	0
345952	744747	Atibaia	9	2013	326	6	m	não reagente		0	0	0
345952	744747	Atibaia	9	2013	327	20	m	não reagente		0	0	0
345952	744747	Atibaia	9	2013	328	15	m	não reagente		0	0	0
345952	744747	Atibaia	9	2013	329	20	m	não reagente		0	0	0
345952	744747	Atibaia	9	2013	330	15	m	não reagente		0	0	0
345952	744747	Atibaia	9	2013	331	22	m	não reagente		0	0	0
345952	744747	Atibaia	9	2013	332	13	m	não reagente		0	0	0
345952	744747	Atibaia	9	2013	333	20	m	não reagente		0	0	0
345952	744747	Atibaia	9	2013	334	18	f	não reagente		0	0	0
345952	744747	Atibaia	9	2013	335	15	m	reagente	1\64	1/128	1/128	
345952	744747	Atibaia	9	2013	336	25	m	não reagente		0	0	0
345952	744747	Atibaia	9	2013	337	15	f	não reagente		0	0	0
345952	744747	Atibaia	9	2013	338	20	m	não reagente		0	0	0
345952	744747	Atibaia	9	2013	339	15	f	não reagente		0	0	0
345952	744747	Atibaia	9	2013	340	25	f	não reagente		0	0	0

345952	744747	Atibaia	9	2013	341	17	f	não reagente	0	0	0
345952	744747	Atibaia	9	2013	342	10	m	não reagente	0	0	0
345952	744747	Atibaia	9	2013	343	12	f	não reagente	0	0	0
345952	744747	Atibaia	9	2013	344	12	m	não reagente	0	0	0
345952	744747	Atibaia	9	2013	345	12	f	não reagente	0	0	0
345952	744747	Atibaia	9	2013	346	10	m	não reagente	0	0	0
345952	744747	Atibaia	9	2013	347	10	m	não reagente	0	0	0
345952	744747	Atibaia	9	2013	348	6	m	não reagente	0	0	0
345952	744747	Atibaia	9	2013	349	9	f	não reagente	0	0	0
345952	744747	Atibaia	9	2013	350	25	m	não reagente	0	0	0
345952	744747	Atibaia	9	2013	351	4	m	não reagente	0	0	0
345952	744747	Atibaia	9	2013	352	15	m	não reagente	0	0	0
345952	744747	Atibaia	9	2013	353	15	m	não reagente	0	0	0
345952	744747	Atibaia	9	2013	354	25	f	não reagente	0	0	0
299264	7465072	Jaguariuna	9	2013	355	13	m	não reagente	0	0	0
299264	7465072	Jaguariuna	9	2013	356	6	f	não reagente	0	0	0
299264	7465072	Jaguariuna	9	2013	357	8	f	não reagente	0	0	0
299264	7465072	Jaguariuna	9	2013	358	10	m	reagente	1\64	1\64	1/128
299264	7465072	Jaguariuna	9	2013	359	9	f	não reagente	0	0	0
299264	7465072	Jaguariuna	9	2013	360	6	f	não reagente	0	0	0
299264	7465072	Jaguariuna	9	2013	361	18	f	reagente	1/128	0	0
299264	7465072	Jaguariuna	9	2013	362	12	f	reagente	1/128	0	0
299264	7465072	Jaguariuna	9	2013	363	7	m	reagente	1/256	0	0
299264	7465072	Jaguariuna	9	2013	364	3	m	reagente	1/256	0	0
299264	7465072	Jaguariuna	9	2013	365	3	m	não reagente	0	0	0
299264	7465072	Jaguariuna	9	2013	366	3	m	reagente	1/256	0	0
299264	7465072	Jaguariuna	9	2013	367	6	m	reagente	1/128	0	1/128
299264	7465072	Jaguariuna	9	2013	368	6	m	não reagente	0	0	0
299264	7465072	Jaguariuna	9	2013	369	3	f	não reagente	0	0	0
299264	7465072	Jaguariuna	9	2013	370	1	f	não reagente	0	0	0
299264	7465072	Jaguariuna	9	2013	371	14	f	reagente	1\64	0	1\64

299264	7465072	Jaguariuna	9	2013	372	8	m	não reagente	0	0	0
299264	7465072	Jaguariuna	9	2013	373	12	m	reagente	1\64	0	0
299264	7465072	Jaguariuna	9	2013	374	15	m	reagente	1\64	0	0
324751	7457449	Braganca Paulista	9	2013	375	17		reagente	1\64	0	0
324751	7457449	Braganca Paulista	9	2013	376	11		reagente	1/128	0	0
324751	7457449	Braganca Paulista	9	2013	377	14		não reagente	1/256	0	0
324751	7457449	Braganca Paulista	9	2013	378	12		não reagente	0	0	0
324751	7457449	Braganca Paulista	9	2013	379	5		reagente	1/256	0	1\64
324751	7457449	Braganca Paulista	9	2013	380	15		reagente	1/256	0	1\64
324751	7457449	Braganca Paulista	9	2013	381	15		não reagente	0	0	0
324751	7457449	Braganca Paulista	9	2013	382	11		reagente	1\1024	1\64	1\64
324751	7457449	Braganca Paulista	9	2013	383	15		reagente	1/256	0	0
324751	7457449	Braganca Paulista	9	2013	384	16		reagente	1/256	0	0
324751	7457449	Braganca Paulista	9	2013	385	19		não reagente	0	0	0
324751	7457449	Braganca Paulista	9	2013	386	11		reagente	1\512	0	0
324751	7457449	Braganca Paulista	9	2013	387	4		não reagente	0	0	0
324751	7457449	Braganca Paulista	9	2013	388	4		reagente	1\64	0	0
324751	7457449	Braganca Paulista	9	2013	389	10		reagente	1/256	0	0
324751	7457449	Braganca Paulista	9	2013	390	10		reagente	1\1024	0	0
324751	7457449	Braganca Paulista	9	2013	391	11		reagente	1\64	0	0
324751	7457449	Braganca Paulista	9	2013	392	20		reagente	1\64	0	0
324751	7457449	Braganca Paulista	9	2013	393	14		não reagente	0	0	0
324751	7457449	Braganca Paulista	9	2013	394	7		não reagente	0	0	0
324751	7457449	Braganca Paulista	9	2013	395	20		não reagente	0	0	0
324751	7457449	Braganca Paulista	9	2013	396	12		não reagente	0	0	0
324751	7457449	Braganca Paulista	9	2013	397	12		não reagente	0	0	0
324751	7457449	Braganca Paulista	9	2013	398	11		não reagente	0	0	0
324751	7457449	Braganca Paulista	9	2013	399	7		não reagente	0	0	0
324751	7457449	Braganca Paulista	9	2013	400	15		reagente	1/256	1/128	1\64
324751	7457449	Braganca Paulista	9	2013	401	10		não reagente	0	0	0
324751	7457449	Braganca Paulista	9	2013	402	22		não reagente	0	0	0

324751	7457449	Braganca Paulista	9	2013	403	15	não reagente	0	0	0
324751	7457449	Braganca Paulista	9	2013	404	6	reagente	1\512	0	0
324751	7457449	Braganca Paulista	9	2013	405	12	reagente	1\64	0	0
324751	7457449	Braganca Paulista	9	2013	406	25	não reagente	0	0	0
324751	7457449	Braganca Paulista	9	2013	407	12	não reagente	0	0	0
324751	7457449	Braganca Paulista	9	2013	408	9	não reagente	0	0	0
324751	7457449	Braganca Paulista	9	2013	409	10	reagente	1/128	0	0
324751	7457449	Braganca Paulista	9	2013	410	9	não reagente	0	0	0
324751	7457449	Braganca Paulista	9	2013	411	8	não reagente	0	0	0
324751	7457449	Braganca Paulista	9	2013	412	5	não reagente	0	0	0
324751	7457449	Braganca Paulista	9	2013	413	12	não reagente	0	0	0
324751	7457449	Braganca Paulista	9	2013	414	9	reagente	1\512	0	0
324751	7457449	Braganca Paulista	9	2013	415	15	não reagente	0	0	0
324751	7457449	Braganca Paulista	9	2013	416	20	não reagente	0	0	0
324751	7457449	Braganca Paulista	9	2013	417	23	reagente	1\64	0	0
324751	7457449	Braganca Paulista	9	2013	418	5	não reagente	0	0	0
324751	7457449	Braganca Paulista	9	2013	419	14	reagente	1/256	0	0
324751	7457449	Braganca Paulista	9	2013	420	9	não reagente	0	0	0
324751	7457449	Braganca Paulista	9	2013	421	7	reagente	1\64	0	0
324751	7457449	Braganca Paulista	9	2013	422	30	não reagente	0	0	0
324751	7457449	Braganca Paulista	9	2013	423	30	reagente	1/256	0	1\64
324751	7457449	Braganca Paulista	9	2013	424	16	reagente	1\64	1\64	0
324751	7457449	Braganca Paulista	9	2013	425	13	reagente	1/256	0	0
324751	7457449	Braganca Paulista	9	2013	426	8	reagente	1/256	0	1/128
324751	7457449	Braganca Paulista	9	2013	427	10	não reagente	0	0	0
324751	7457449	Braganca Paulista	9	2013	428	7	não reagente	0	0	0
324751	7457449	Braganca Paulista	9	2013	429	13	reagente	1\64	1\64	0
324751	7457449	Braganca Paulista	9	2013	430	6	reagente	1\512	0	0
324751	7457449	Braganca Paulista	9	2013	431	6	não reagente	0	0	0
324751	7457449	Braganca Paulista	9	2013	432	14	não reagente	0	0	0
324751	7457449	Braganca Paulista	9	2013	433	11	não reagente	0	0	0

324751	7457449	Braganca Paulista	9	2013	434	17		reagente	1\64	0	1\64
324751	7457449	Braganca Paulista	9	2013	435	15		reagente	1/256	0	0
324751	7457449	Braganca Paulista	9	2013	436	8		reagente	1/256	0	0
324751	7457449	Braganca Paulista	9	2013	437	8		reagente	1\64	0	1\64
324751	7457449	Braganca Paulista	9	2013	438	12		reagente	1\512	0	0
324751	7457449	Braganca Paulista	9	2013	439	18		reagente	1/256	1\64	0
324751	7457449	Braganca Paulista	9	2013	440	12		reagente	1\1024	0	0
220336	7490031	Piracicaba	11	2013	441	7	m	não reagente	0	0	0
220336	7490031	Piracicaba	11	2013	442	10	f	não reagente	0	0	0
220336	7490031	Piracicaba	11	2013	443	6	f	reagente	1\256	0	1\64
220336	7490031	Piracicaba	11	2013	444	10	f	não reagente	0	0	0
228943	7486163	Piracicaba	12	2013	445	25	f	não reagente	0	0	0
228943	7486163	Piracicaba	12	2013	446	20	f	reagente	1\128	0	1\256
228943	7486163	Piracicaba	12	2013	447	30	m	reagente	1\512	1\64	1\64
228943	7486163	Piracicaba	12	2013	448	9	m	reagente	1\64	1\256	1\128
228943	7486163	Piracicaba	12	2013	449	25	f	não reagente	0	0	0
228943	7486163	Piracicaba	12	2013	450	18	m	não reagente	0	0	0
228943	7486163	Piracicaba	12	2013	451	18	m	não reagente	0	0	0
228943	7486163	Piracicaba	12	2013	452	10	m	não reagente	0	0	0
228943	7486163	Piracicaba	12	2013	453	10	m	reagente	1\128	1\256	0
230739	7486685	Piracicaba	12	2013	454	8	f	reagente	1\128	0	1\64
230739	7486685	Piracicaba	12	2013	455	3	m	reagente	1\64	0	0
230739	7486685	Piracicaba	12	2013	456	2	m	reagente	1\256	0	0
230739	7486685	Piracicaba	12	2013	457	4	m	não reagente	0	0	0
230739	7486685	Piracicaba	12	2013	458	6	m	reagente	1\64	1\256	0
230739	7486685	Piracicaba	12	2013	459	5	f	não reagente	0	0	0
230739	7486685	Piracicaba	12	2013	460	3	f	não reagente	0	0	0
230739	7486685	Piracicaba	12	2013	461	14	m	não reagente	0	0	0
		Piracicaba	12	2013	462	16	m	não reagente	0	0	0
		Piracicaba	12	2013	463	10	m	reagente	1\64	1\128	1\64
		Piracicaba	12	2013	464	10	m	reagente	1\64	0	0

		Piracicaba	12	2013	465	8	f	reagente	1\128	1\64	0
		Piracicaba	12	2013	466	5	m	não reagente	0	0	0
		Piracicaba	12	2013	467	11	m	não reagente	0	0	0
		Piracicaba	12	2013	468	8	f	reagente	1\512	0	1\256
		Piracicaba	12	2013	469	10	m	reagente	1\512	0	0
		Piracicaba	12	2013	470	10	m	reagente	1\512	1\256	0
		Piracicaba	12	2013	471	9	m	reagente	1\128	1\512	0
		Piracicaba	12	2013	472	8	f	não reagente	0	0	0
		Piracicaba	12	2013	473	10	f	reagente	1\64	1\128	0
		Piracicaba	12	2013	474	10	m	reagente	1\64	0	0
		Piracicaba	12	2013	475	15	f	reagente	1\64	1\64	0
		Piracicaba	12	2013	476	17	f	reagente	1\64	0	0
		Piracicaba	12	2013	477	1	f	não reagente	0	0	0
		Piracicaba	12	2013	478	6	m	reagente	1\64	1\128	0
		Piracicaba	12	2013	479	10	m	reagente	1\64	0	1\256
		Piracicaba	12	2013	480	3	m	não reagente	0	0	0
		Piracicaba	12	2013	481	3	m	não reagente	0	0	0
		Piracicaba	12	2013	482	7	m	reagente	1\64	0	0
		Piracicaba	12	2013	483	8	m	reagente	1\64	0	0
		Piracicaba	12	2013	484	8	m	não reagente	0	0	0
		Piracicaba	12	2013	485	9	f	não reagente	0	0	0
		Piracicaba	12	2013	486	9	f	reagente	1\64	1\256	1\64
		Piracicaba	12	2013	487	4	f	reagente	1\64	0	0
232087	7481315	Piracicaba	1	2014	488	3	f	reagente	1/512	0	1\64
232087	7481315	Piracicaba	1	2014	489	15	m	reagente	1/256	0	1\128
232087	7481315	Piracicaba	1	2014	490	20	m	não reagente	0	0	0
232087	7481315	Piracicaba	1	2014	491	4	f	não reagente	0	0	0
232087	7481315	Piracicaba	1	2014	492	20	f	reagente	1/128	0	0
232087	7481315	Piracicaba	1	2014	493	15	f	não reagente	0	0	0
232087	7481315	Piracicaba	1	2014	494	10	f	reagente	1/512	0	1\64
232087	7481315	Piracicaba	1	2014	495	5	m	não reagente	0	0	0

232087	7481315	Piracicaba	1	2014	496	14	m	não reagente		0	0	0
232087	7481315	Piracicaba	1	2014	497	2	m	não reagente		0	0	0
232087	7481315	Piracicaba	1	2014	498	5	m	reagente	1/128		0	0
232087	7481315	Piracicaba	1	2014	499	12	m	reagente	1/256		0	0
232087	7481315	Piracicaba	1	2014	500	18	f	reagente	1/256		0	1024
232087	7481315	Piracicaba	1	2014	501	14	m	não reagente		0	0	0
232087	7481315	Piracicaba	1	2014	502	8	f	reagente	1\64		0	1/512
232087	7481315	Piracicaba	1	2014	503	15	f	reagente	1\64		0	1/256
234515	7488107	Piracicaba	12	2013	504	12	m	não reagente		0	0	0
